# Improving the Accessibility and Efficiency of Point-of-Care Diagnostics Services in Low- and Middle-Income Countries: Lean and Agile Supply Chain Management

**DOI:** 10.3390/diagnostics7040058

**Published:** 2017-11-29

**Authors:** Desmond Kuupiel, Vitalis Bawontuo, Tivani P. Mashamba-Thompson

**Affiliations:** 1 Department of Public Health Medicine, School of Nursing and Public Health, University of KwaZulu-Natal, 4001 Durban, South Africa; Mashamba-Thompson@ukzn.ac.za; 2 Faculty of Health and Allied Sciences, Catholic University College of Ghana, Fiapre, Sunyani, Ghana; bawontuovitalis@yahoo.com

**Keywords:** accessibility, point-of-care diagnostics, low- and middle-income countries

## Abstract

Access to point-of-care (POC) diagnostics services is essential for ensuring rapid disease diagnosis, management, control, and surveillance. POC testing services can improve access to healthcare especially where healthcare infrastructure is weak and access to quality and timely medical care is a challenge. Improving the accessibility and efficiency of POC diagnostics services, particularly in resource-limited settings, may be a promising route to improving healthcare outcomes. In this review, the accessibility of POC testing is defined as the distance/proximity to the nearest healthcare facility for POC diagnostics service. This review provides an overview of the impact of POC diagnostics on healthcare outcomes in low- and middle-income countries (LMICs) and factors contributing to the accessibility of POC testing services in LMICs, focusing on characteristics of the supply chain management and quality systems management, characteristics of the geographical location, health infrastructure, and an enabling policy framework for POC diagnostics services. Barriers and challenges related to the accessibility of POC diagnostics in LMICs were also discussed. Bearing in mind the reported barriers and challenges as well as the disease epidemiology in LMICs, we propose a lean and agile supply chain management framework for improving the accessibility and efficiency of POC diagnostics services in these settings.

## 1. Introduction

Globally, point-of-care (POC) diagnostics are essential for ensuring rapid diagnosis and disease management, and for disease surveillance and control programs [1,2]. Diagnostics development has been delayed for several neglected diseases, and even where good tests are available, they are not necessarily accessible or affordable to those who need them most [3,4]. It is estimated that, out of about 9 million tuberculosis (TB) cases each year, over 2.5 million cases are either not diagnosed or not notified, and only about a third of multidrug-resistant tuberculosis (MDR-TB) patients are diagnosed [3]. More than 50% of people living with HIV are also unaware of their status [3,5]. It is also estimated that, more than 80% of malaria patients are treated based on signs and symptoms in nearly 50% of malaria-endemic low- and middle-income countries (LMICs) [3].

Rapid progress has been made towards POC diagnosis of diseases in LMICs owing to increased investment, technological advances, and greater awareness of the significance of reliable diagnostic tests in recent years [4]. POC diagnostics refers to innovative medical technologies used for testing infectious diseases or medical conditions [2,6,7]. POC testing is a diagnostic test that is conducted near a patient/client and leads to rapid clinical decisions [2,6]. POC testing can shorten the time of clinical decision-making about additional testing or therapy, as delays are no longer caused by transport and preparation of clinical samples, and biochemical test results are rapidly available at the point of care [8]. Studies have shown that POC testing improves medical outcome and lowers the costs of seeking healthcare services in resource-limited settings [8,9,10,11]. 

POC testing has also been proven to aid in the management of patients in emergency departments [12]. For example, POC testing in emergency departments may result in timely discharge of patients, expediting triage of patients, and early treatment initiation, as well as alleviate the negative impacts of overcrowding [12]. Studies have demonstrated that POC testing has the potential to reduce the time to obtain test results and accelerate the diagnosis and initiation of treatment, particularly where healthcare infrastructure is weak and access to quality and timely medical care is a challenge [2,6,13,14]. Other studies have also proven that rapid POC testing delivers prompt results as well as early referrals when compared to standard laboratory testing [4,6,15]. Access to rapid and simple POC diagnostics services in healthcare facilities that lack or have poor laboratory infrastructure and inadequately skilled healthcare workers can improve health outcomes in those areas. Luppa et al. provided an in-depth review of POC testing to address clinically important analytical techniques, organizational concepts, clinical applications, and trends in healthcare [8,16]. However, this review provides an overview of the impact of POC diagnostics on healthcare outcomes and factors that contribute to the accessibility of POC diagnostics in LMICs, such as characteristics of the supply chain management and quality management systems, characteristics of the geographical location, health infrastructure, and an enabling policy framework for POC diagnostics services. Barriers and challenges related to the accessibility of POC diagnostics services in LMICs were also discussed.

## 2. Impact of POC Diagnostics on Health Outcomes in LMICs

The advent of point-of-care (POC) diagnostics has a transformative effect on healthcare in LMICs, including the management of medical conditions and obstetric complications associated with pregnancy [4,16]. For instance, Mashamba-Thompson et al. demonstrated the impact of rapid HIV testing on maternal health outcomes [14]. The study showed the significant effect of HIV POC diagnostics on the following: decreased mother-to-child transmission of HIV with an ES of 0.86 (95% CI, 0.79–0.93); increased linkage to both antiretroviral treatment and HIV care for infected women with an ES of 0.76 (95% CI, 0.69–0.84); and ES of 0.50 (95% CI, 0.18–0.82) respectively [14]. Also, access to improved prenatal syphilis POC testing enabled same-day testing and treatment for pregnant women and their partners, which positively impacted on newborn health [17,18,19]. In addition, implementation of high-quality dual POC tests for HIV and syphilis resulted in substantially increased coverage and uptake for antenatal syphilis screening and prevented more than 290,000 adverse pregnancy outcomes owing to syphilis yearly [17,20,21,22,23,24]. POC CD4+ T-cell enumeration has resulted in a decreased time (44 days to 17 days) for initiation of antiretroviral therapy among women eligible for treatment, as well as an overall increase in hemoglobin and syphilis testing from 67.9% to 83% and 80.8% to 87%, respectively [16].

Furthermore, POC testing services have an impact on disease surveillance and control. An increased rate of HIV and malaria testing and case detection catalyzed by rapid POC testing have driven global efforts in HIV and malaria prevention and treatment in LMICs [25]. POC testing for sexually transmitted infection (STI) has expanded screening and reduced syndromic management of STIs and loss to follow-up [26]. STI POC testing has also expedited treatment and partner care, created opportunities for new social and financial models of community-based testing services, and increased equity and access to testing [26]. Branson et al. reported that POC tests have made HIV testing accessible in areas with limited laboratory facilities and significantly reduced the number of people who are unaware of their status [27].

## 3. Efficient Supply Chain Management for POC Diagnostics in LMICs

Efficient supply chain management ensures adequate access to reliable POC diagnostics for healthcare professionals [1,28]. Supply chain management of POC diagnostics involves all the processes or events extending from the identification of a client’s need through to the production, selection, quantification, negotiation with suppliers, procurement, quality assurance, storage, inventory management, distribution and redistribution, usage, and safe disposal of used POC testing kits [29,30]. Strict adherence to supply chain management procedures guarantees the accessibility of POC testing services [30]. Encouraging regional or district distribution hubs closer to end users is critical to prevent testing delays due to test kits being out of stock [31]. Also, employing qualified and well-paid supply chain management personnel in purchasing services or managing the supply chain of POC diagnostics, ensures sustainability of the service across all level of healthcare [32]. Hinrichs et al. reported that, efficient procurement and potential cost savings may be achieved through shared approaches to purchasing, improving relations with suppliers, building competences and abilities for purchasing decisions, and the use of technology for data and materials management [33]. 

Selection and procurement of POC diagnostics test in accordance with national guidelines and laboratory strategic plans as part of the supply and demand value chain are vital and ensures access to POC testing [1,31,34]. Adherence to national guidelines and laboratory strategic plans for the supply of POC diagnostics is imperative as the procurement market in LMICs can be unregulated [31,35]. Donated POC diagnostics are sometimes not in line with a country’s needs yet it can be difficult to reject donors’ offerings [31,35]. Again, the selection of POC diagnostics in accordance with the World Health Organization (WHO) quality-ASSURED (Affordability, Sensitivity, Specificity, User-friendly, Rapid and robust, Equipment-free and Delivered) criteria for rural and remote clinics [2,36,37], as part of supply chain management, not only ensures the accessibility, availability, and use of POC diagnosis, but also improves access to healthcare.

## 4. Quality Systems Management for POC Diagnostics in LMICs

Implementation of comprehensive quality management of POC testing services is of the utmost importance in ensuring the accessibility of the service [38,39,40]. Fonjungo et al. defined quality management systems as including leadership, training, appropriate quality control, standardized management tools, new lot validation, site supervision, and external quality assessment [31,40]. Quality management systems enable the identification of a facility and diagnostics or assay-associated errors for corrective actions, and thus translate to accessibility of POC testing services, especially in LMICs, where traveling to central laboratories for testing is common [30,31,39,40]. Proficiency testing programs should be an integral component of all forms of testing to ensure confidence in testing results and improve patient care and health outcomes [39]. For POC testing to reach its fullest potential in LMICs, disposables need to be robust, allowing for shipping and storage at the required temperature; reagents and antibodies for immunoassays must be stored in a disposable way such as in a lyophilized state in vapor-proof compartments to prevent degradation [30,31,41]. Data connectivity further allows disease control programs to monitor the quality of POC testing and optimize supply chain management, thereby increasing the efficiency of healthcare management systems and improving patient outcomes [9,30,42]. According to Alemnji et al., the development of a functional laboratory network that facilitates communication, referral testing, training, and quality improvement among laboratories and testing sites is vital for the overall improvement of laboratory services beyond HIV [39].

## 5. Geographical Considerations Regarding Healthcare Access for POC Diagnostics in LMICs

Accessibility of POC testing in terms of place can be defined as the proximity to the nearest healthcare facility for POC diagnostics services [43,44]. In other words, accessibility of healthcare refers to the relative ease with which services can be reached from a given location [45]. Accessibility of healthcare generally encompasses both spatial and non-spatial factors [44,46,47]. Spatial access emphasizes the importance of spatial separation between supply such as healthcare providers and demand (population) and how they are related in space [44,47,48]. Hence, spatial access is determined by where one is located [44,45]. Non-spatial factors of healthcare accessibility may include demographic and socioeconomic variables such as social class, income, age, sex, and race, which also interact with spatial access [47,49]. Equity of access to healthcare is assumed to be when healthcare services are distributed on the basis of people’s need [47]. For the patient, the ability to obtain POC testing, diagnosis, and treatment in a single visit to the nearest health facility is comforting because it saves patient transport cost, time lost from work, and the stress of having to return for test results later [30,44]. For this reason, Peeling and colleagues indicated that the selection and procurement of a POC diagnostic should, among other factors, take into consideration the geographical location (rural/urban) in which the test kits will be used [4,44]. This supports one of the core values of the Alma-Ata declaration, which emphasizes the use of appropriate technology that is affordable, relevant to the needs of the population, and scientifically sound [50,51]. This declaration potentially enhances access to POC diagnostics services, particularly in rural communities.

## 6. Health Infrastructure for POC Diagnostics in LMICs

Availability of adequate healthcare infrastructure including strong laboratory systems as well as storage facilities and a regular supply of electricity form an integral part of providing POC testing services. However, adopting available rapid POC diagnostics requiring no electricity is essential for ensuring POC testing at the primary healthcare level where there is a lack of laboratory infrastructure [2,4,34]. Studies have shown that, generally, access to laboratory services in LMICs is inadequate and hence the creation of an essential diagnostics list (EDL) for in vitro diagnostics (IVD) by WHO helps increase access to POC testing in rural and remote areas [52,53,54]. Fonjungo et al. proposed that POC testing should be implemented as part of the existing laboratory networks, and, in some cases, may serve as a temporary backup for laboratory-based testing sites in the event of equipment malfunction or shortage of reagents [30]. Connecting POC testing devices, where possible, with wireless or Web-based systems allows them to be used to monitor and transmit results, error rates, and reagent consumption from lower health facilities to a district, regional, or central laboratory, with feedback or planned corrective actions provided in real time [25,42,55]. Connectivity can enhance the monitoring of test results and create a real-time information flow for the use of POC devices to improve quality [30,42].

## 7. Enabling Policy Framework for POC Diagnostics in LMICs

The availability of an enabling policy framework for POC diagnostics services could potentially improve POC testing in LMICs. Clearer national policies in terms of POC diagnostics evaluation and certification, financing, training and expertise, as well as research is crucial to guide the implementation of POC testing in LMICs [3,30]. Addressing the regulatory challenges that result in delayed approval of POC testing increases accessibility and improves healthcare outcomes [30,42]. Also, the availability of national policy guidelines/regulations on supply chain management of POC diagnostics ensures consistency of supply, appropriate use, and quality testing [3,30,34,56]. In addition, studies have demonstrated that an adequate national policy plan for POC diagnostics supply chain management and quality systems management prior to the implementation of a POC testing program improves accessibility [30,34,36,42,56]. In addition, the availability of guidelines for the POC diagnostics approval process and post-market surveillance, biosafety, an appropriate body/department responsible for standard regulatory issues, and human resources training enhances access to POC testing [30,42,56]. For instance, using a standard tool for personnel training enhances the skills of both laboratory and non-laboratory testing personnel, as well as other clinicians such as medical doctors and nurses/midwives who request and act upon test results [57]. Comprehensive practical training and assessment of personnel for POC testing competency and certification, including certification of health facilities for POC testing services for a valid duration, improves access to healthcare, particularly in LMICs [30]. 

## 8. Barriers and Challenges Related to Accessibility of POC Diagnostics in LMICs

Despite the recent increase in investment in POC diagnostics for LMICs, numerous barriers prevent the adoption of existing diagnostics and the development and introduction of new diagnostics in LMICs [4,56,58]. Barriers related to accessibility of POC diagnostics in LMICs include: health system and infrastructure barriers, geographical barriers of accessibility, supply chain issues, and research-related barriers. Challenges to the implementation of POC diagnostics in LMICs also include the following: policy/regulatory guidelines and funding challenges and challenges relating to POC diagnostics development in LMICs. Generally, constraints such as financial, human resources, policy regulatory, infrastructure, quality control and quality assurance, work-flow balance, training of personnel, supply chain, infection risk, administrative, technical awareness, health systems problems, and the relationship between healthcare workers and patients have been identified by various studies [3,4,9,34,42,56,59,60,61]. Pai et al. observed that barriers to POC testing in LMICs may differ from one country to another across public and private sectors and urban and rural settings, and there may be disease-specific barriers to some diagnostics [2].

### 8.1. Policy/Regulatory Guidelines and Funding Challenges

Many countries lack regulations/policies for POC diagnostics, which may result in widespread use of sub-standard POC diagnostics for testing various medical conditions; this makes it difficult for manufacturers [4,34,56,62]. Fragmented, unclear, and complex regulatory and registration processes at both international and national levels have made it difficult to map the most effective route to product registration when targeting a multi-country market for diagnostics [34,56]. Many LMICs also lack established criteria or guidelines for licensing and introducing new diagnostic tests [2,4,56,62]. Poor regulation of diagnostics may result in easy availability of suboptimal and poor-quality rapid tests on the market, which in turn make it challenging to scale up prequalified POC diagnostic tests in LMICs [2,56]. Studies have found that weak regulations on diagnostics are more challenging compared to for drugs and vaccines [3,4,56,62]. 

Again, the implementation of POC diagnostics services in LMICs presents a logistical challenge beyond the additional cost for services, maintenance, and repairs [34,56]. The cost of POC testing is frequently underestimated when the additional costs of implementation, staffing, training, and maintenance are excluded from “cost per test” calculations [3,34]. However, a major barrier is the inconsistent funding of diagnostic tests in most LMICs by government and donors, leaving these countries to rely heavily on development partners, non-governmental organizations (NGOs), and multinational organizations for assistance [3,34,56]. For example, HIV/AIDS programs in some countries in Sub-Saharan Africa are solely funded by external sources such as grants from the U.S. President's Emergency Plan for AIDS Relief (PEPFAR) and the Global Fund to Fight AIDS, Tuberculosis, and Malaria [63]. 

### 8.2. Health System and Infrastructure Barriers

Successful implementation of POC testing further requires the availability of adaptable diagnostic technologies, improved platforms and back-up infrastructure, trained human resource and key stakeholders engagement, including public health representatives, developers, suppliers, healthcare professionals, local health authorities, and the community [56,64]. Competent healthcare workers are essential for the delivery of health interventions including POC testing services [31,56,65]. However, LMICs are saddled with shortages of skilled healthcare professionals, and the few skilled healthcare workers are overburdened with a high volume of patients; work-flow and time constraints do not permit easy use of POC tests in these countries [2,3,56]. Unqualified and casual healthcare workers may lack the knowledge and training needed to implement even simple rapid diagnostic tests (RDTs). Therefore, inaccurate results may erode the health system’s faith or cause clinicians to become disillusioned with POC testing, and many clinicians may rely on clinical judgment for clinical decisions [2,4,34]. In addition to weak health systems and non-functional diagnostic systems, problems with infrastructure, end-user knowledge, and utility are also challenging [2,3,34,56]. For example, primary healthcare clinics in rural areas often lack infrastructure such as constant power supply, refrigerators, storage space, waste disposal units, phlebotomy supplies, and temperature control; this makes it hard to implement some types of POC tests in these settings [2]. Poor laboratory infrastructure poses a barrier to scaling up such POC testing technologies [2,37,56]. Laboratory professionals in hospitals and larger healthcare facilities are most often opposed to any testing done outside the laboratory setting due to fear of its implications for their own business, and they also worry about relinquishing control over testing [2,56]. 

### 8.3. Challenges with POC Diagnostics Development

Major challenges also exist for the biomedical engineering community in developing diagnostic tests suitable for resource-limited settings in the developing world [55,56,66]. Biomedical engineers have traditionally developed technologies in response to the needs of developed countries that have met the requirements of well-funded laboratories in highly regulated and quality-assessed environments [55,66]. This approach, however, does not address the needs of the majority of people suffering with infectious diseases in the world with limited access to resourced healthcare facilities with almost no supporting clinical laboratory infrastructure [34,55,56]. Different design criteria must be taken into consideration when designing POC diagnostics for centralized testing in a national laboratory versus regional/provincial, district, and rural health clinics in a remote setting with no or poor infrastructure [56,67]. Available tests may not quite meet user needs since rapid tests are most often focused on a single disease, whereas primary healthcare workers are more worried about syndromes of unknown etiology [2]. There are challenges with local production versus imported diagnostics apart from cost-based and reimbursement limitation incentives to develop POC diagnostics, and compromised training due to lack of infrastructure, technologies, and material for diagnostics production [3,56]. 

### 8.4. Geographical Barriers to Access

Geographical access is also a major barrier to POC diagnostics but hardly audited despite its importance in healthcare access, hence preventing integration in national-level health system assessment and planning [44,68]. A significant proportion of the population is rural-based in LMICs and access to healthcare facilities means walking long distances or traveling by public transport, with its related cost and stigma [39,69,70]. For instance, Gething et al. reported that a third of women in Ghana live beyond the clinically significant two-hour threshold from facilities likely to offer emergency obstetric and neonatal care (EmONC) [68]. Again, access to POC HIV testing and treatment in Sub-Saharan Africa is compromised by a variety of factors, including distance to health facilities and multiple, conflicting financial burdens [44,69]. 

### 8.5. Supply Chain Barriers

One of the greatest limitations of the most widely used POCT, the HIV rapid test, is the supply chain [31]. The supply chain poses a major potential barrier to the accessibility of POC testing, particularly in resource-limited settings [18,19,39,42,56,61,71,72,73,74,75]. Irregular supply, poor forecasting, selection of appropriate diagnostics, unclear procurement systems, delay distribution systems, poor maintenance of quality assurance, and inadequate stock affect existing diagnostics [3,56]. Supply chain deficiencies can lead to suboptimal or poor-quality POC tests, which, in turn, may discredit POC testing [2]. Biza et al. identified a poor supply chain as a major barrier to the implementation of antenatal care packages in three health facilities in Mozambique [76]. Robust procurement systems are vital to effectively implement new POC technologies, especially when reagents and quality control panels have limited shelf lives and frequently require cold-chain management [2,3,34,56]. Multiple potential bottlenecks also exist in the implementation of effective POC diagnostics distribution systems and subsystems such as a lack of human resources, weak information management systems to track inventory, overburdened healthcare facilities, and slow administrative processes for requisitions of laboratory tests [3,34,56]. Maintenance of quality can also present a challenge for healthcare workers in LMICs due to lack of expertise or training [34,56]. Stevens et al. recognized a sustainable supply chain and reimbursement strategies as additional requirements for HIV management [40]. Thairu et al. advised that supply chain, corporate commitment to implementation, and community factors require consideration when adopting newer and less expensive technologies such as POC diagnostics to facilitate access to CD4 testing [61]. Inconsistency in purchasing practices from the donor community and national programs also leads to ambiguity in targeting price points and product specification trade-off decisions and, therefore, excludes the end user and patient/client as an important stakeholder in defining product attributes and adoption decisions [34]. Engel et al. also identified a lack of end-user involvement in research and development of POC diagnostics, as well as insufficient evaluation of POC diagnostic in settings of intended use of a diagnostic test as key barriers in the POC diagnostic value chain [56]. In addition, lack of local and multidisciplinary capacity for operational research in POC diagnostics is a challenge in POC diagnostics value chain system [3,56]. 

## 9. Discussion

The benefits of improved accessibility to POC diagnostics services are significant. This review demonstrates the impacts of POC testing services such as enabling rapid diagnosis and informing prompt clinical decisions [4,6,11,12,14,16,17,20,21,25,26,27,77,78,79]. It also reveals that successful implementation of POC diagnostics services requires appropriate healthcare infrastructure, efficient supply chain management, quality systems management, enabling a policy framework and as well as geographical consideration during implementation [38,40,56,64]. Despite the significant impact and increase in development of POC diagnostics in recent times [4,6], the literature demonstrates that access to POC testing remains a challenge requiring urgent attention. Moreover, the literature shows some potential challenges and barriers that impede accessibility. These include: health system and infrastructure, geographic access, supply chain and research-related barriers, as well as policy, funding, and POC diagnostics development challenges in LMICs [2,3,4,34,56]. In addition, this review proves that these barriers and challenges do not act in isolation and require the attention of all stakeholders in order to improve access to POC diagnostic services. 

The World Health Organization (WHO) has developed an essential diagnostics list (EDL) focusing on the need to improve access to healthcare [54]. It is estimated that HIV/AIDS spending in 2031 will be 1–3% of gross domestic product (GDP) for high-burden, low-income (HBLI) countries [63]. A further 23–65% expenditure of GDP on healthcare is expected within the same period, suggesting that HBLI countries will be relying on external financing for HIV/AIDS for several decades to come [63]. However, studies have recommended that the selection of POC testing for rural clinics should follow the WHO quality-ASSURED criteria [2,36,37]. This may help procure the needed POC tests and save money. 

As demonstrated in this review, supply chain management is a major barrier to the accessibility, availability, and use of POC diagnostics [2,3,4,34,56]. Chandra and Grabis reiterated that an agile, responsive and flexible POC supply chain should be characterized by customer-oriented networking and flexible supply chains [80]. This includes introduction of new product(s), or upgrade of existing product(s); introduction of new, or improvement of existing, process(es); allocation of new, or re-allocation of existing resource(s); selection of new supplier(s), or deselection of existing ones; changes in demand patterns for product(s) manufactured; changes in lead times for product and or process life cycles; and changes in commitments within or between supply chain members [80].

**Figure 1:**
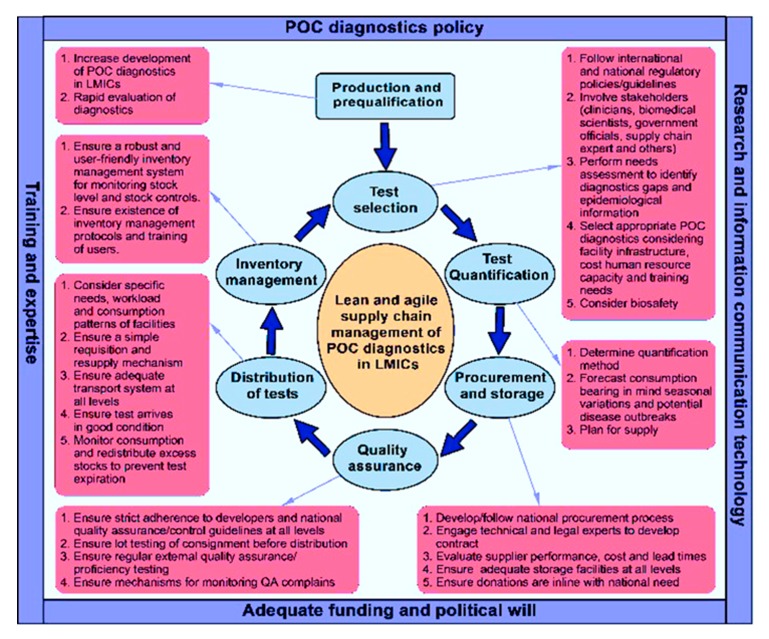
Lean and agile supply chain management framework for improving access to POC diagnostics services in LMICs.

This review demonstrates that the accessibility and efficiency of POC diagnostics services have major implications on healthcare outcomes. Supply chain management poses a major threat to the accessibility of POC diagnostics services, particularly in hard-to-reach populations [18,19,39,42,56,61,71,72,73,74,75]. Taking into consideration the economic condition, level of infrastructure, human resource capacity, and other resources, as well as the high disease burden in the majority of LMICs, we propose a lean and agile supply chain management framework to ensure sustainable access to POC diagnostics services in LMICs (Figure 1).

A lean and agile supply chain management framework for POC diagnostics can potentially respond to patients’/clients’ changing testing needs efficiently and effectively, whether adopting new or scaling up existing POC diagnostics services. The building blocks of this framework include production and prequalification, selection, quantification, procurement and storage, quality assurance, distribution and redistribution, and inventory management [1,30,81]. These building blocks are further anchored by four main pillars as follows: POC diagnostics policy, adequate funding and political will, training and expertise, and research and information communication technology. The design focused on reducing lead times and streamlining the processes within the POC diagnostics supply chain, bearing in mind the economic challenges and disease burden that often confront LMICs. In addition, it seeks to eliminate waste and non-value-added activities in the supply process, improving access to POC diagnostics services in these settings.

The production of POC diagnostics in LMICs is essential to sustaining the supply chain of POC tests [3,56]. The majority of POC diagnostics undergoing development or produced of late are targeted for use in LMICs [2,34]. However, POC diagnostics production/development is mostly carried out in high-income countries (HICs) [3]. This comes with an additional cost of having to import these diagnostics tests from HICs. In view of this, our framework proposes increased production of POC diagnostics in LMICs alongside rapid evaluation in order to sustain the demand and supply side of POC testing services in rural and remote settings. LMICs could additionally benefit from the business side that comes along with POC diagnostics development. Test selection must be in alignment with national or WHO guidelines. Again, test selection must follow a rigorous assessment involving key stakeholders such as clinicians, biomedical scientists/laboratory workers, supply chain officers, and governmental authorities. This process ensures that appropriate tests are selected for different settings considering epidemiological information such as disease prevalence and morbidity, and also multiplex POC diagnostics for syndromes where necessary [41,82]. Engaging stakeholders not only ensures commitment across all levels and acceptability for users, but also helps in forecasting consumption considering potential increases in usage due to seasonal variation, epidemics, and procurement of tests. The remaining building blocks for this framework are summarized in Figure 1.

Information communication technology is one of the four pillars that this framework is anchored on. This is a result of the recent increase in mobile healthcare, which has transformed the production of next-generation healthcare technologies [8,15,83]. Vashist et al. reported that there are a wide range of prospective assay formats, including bioanalytical platform, diagnostic readers, and technologies that have been interfaced with mobile technologies [15,83]. Interestingly, it is also estimated that over 70% of the 7.4 billion mobile phone users are in LMICs [83]. This increase in mobile phone accessibility provides a great opportunity to improve access to healthcare, including access to POC diagnostics in LMICs. 

Finally, POC diagnostics supply chain in LMICs is mostly integrated into traditional laboratory policies. This often results in very little attention to POC diagnostics services, especially for primary healthcare facilities located in rural communities, where the majority of the population dwells [84,85]. In addition, studies have raised concerns about the rising cost of POC diagnostics development, delayed reimbursement of POC developers by the government and insurance companies, and regulatory challenges that require international standardization and harmonization [3,31,56]. In light of this, a national policy focusing on POC diagnostics with adequate funding and political commitment, and adequate training and expertise with regards to POC development and usage are desirable for lean and agile supply chain management [2,34,69]. In addition, improving and providing funding for research in POC diagnostics-related fields, creating an enabling business environment for POC diagnostics development in LMICs as well as supportive information communication technology, are vital.

## 10. Conclusions

The impact of POC testing in LMICs cannot be underestimated. In view of this, regular assessment of the supply chain management for POC diagnostics services, particularly primary healthcare (PHC) clinics, is needed. This can contribute to a continual improvement of POC diagnostics services, especially for pregnant women receiving antenatal care in rural healthcare facilities. To achieve this, an evidence-based research intervention is urgently needed. Primary studies to assess the accessibility of POC diagnostics services as well as the supply chain management of POC diagnostics in rural PHC clinics in LMICs are recommended. This will aid in identifying practical, country-specific challenges relating to POC diagnostics accessibility and supply chain management and bring them to the attention of policy-makers and relevant health authorities in order to improve access to healthcare to all who need it, as recommended by the World Health Organization. Finally, bearing in mind the recent rise in mobile phone usage in LMICs, feasibility studies to assess the effectiveness of delivering mobile health-linked POC testing are encouraged. This has the potential to facilitate access to POC testing in hard-to-reach populations.
